# Evidence that hematopoietic stem cell function is preserved during aging in long-lived S6K1 mutant mice

**DOI:** 10.18632/oncotarget.8729

**Published:** 2016-04-13

**Authors:** Colin Selman, Amy Sinclair, Silvia M.A. Pedroni, Elaine E. Irvine, Alison M. Michie, Dominic J. Withers

**Affiliations:** ^1^ Glasgow Ageing Research Network (GARNER), Institute of Biodiversity, Animal Health and Comparative Medicine, College of Medical, Veterinary and Life Sciences, University of Glasgow, Glasgow, UK; ^2^ Metabolic Signaling Group, Medical Research Council Clinical Sciences Centre, Imperial College, London, UK; ^3^ Institute of Cancer Sciences, College of Medical, Veterinary and Life Sciences, University of Glasgow, Glasgow, UK

**Keywords:** mammalian target of rapamycin, mTOR, HSC, aging, S6K1, Gerotarget

## Abstract

The mechanistic target of rapamycin (mTOR) signalling pathway plays a highly conserved role in aging; mice lacking ribosomal protein S6 kinase 1 (*S6K1^−/−^*) have extended lifespan and healthspan relative to wild type (WT) controls. Exactly how reduced mTOR signalling induces such effects is unclear, although preservation of stem cell function may be important. We show, using gene expression analyses, that there was a reduction in expression of cell cycle genes in young (12 week) and aged (80 week) *S6K1^−/−^* BM-derived c-Kit^+^ cells when compared to age-matched WT mice, suggesting that these cells are more quiescent in *S6K1^−/−^* mice. In addition, we investigated hematopoietic stem cell (HSC) frequency and function in young and aged *S6K1^−/−^* and WT mice. Young, but not aged, *S6K1^−/−^* mice had more LSK (lineage^−^, c-Kit^+^, Sca-1^+^) cells (% of bone marrow (BM)), including the most primitive long-term repopulating HSCs (LT-HSC) relative to WT controls. Donor-derived engraftment of LT-HSCs in recipient mice was unaffected by genotype in young mice, but was enhanced in transplants using LT-HSCs derived from aged *S6K1^−/−^* mice. Our results are the first to provide evidence that age-associated HSC functional decline is ameliorated in a long-lived mTOR mutant mouse.

## INTRODUCTION

Aging can be slowed and late-life health maintained through environmental, genetic and pharmacological interventions [1]. The current challenge in aging research is to identify exactly how mechanistically these interventions act to elicit their beneficial effects, and critically whether these mechanisms are conserved across different interventions. Stem cell dysfunction is a proposed candidate hallmark of aging [2], and it is well established that stem cells are critical to cellular homeostasis during aging and disease [3]. However, stem cells undergo aging-related changes; hematopoietic stem cells (HSC) exhibit impaired self-renewal potential, stress resistance, engraftment and homing ability during aging [3, 4], all associated with immunosenescence, myelo-proliferative and autoimmune disease [5]. Several studies have investigated whether stem cell function during aging is slowed in long-lived animal models. In male *Drosophila*, dietary restriction(DR) attenuated an age-related loss of germline stem cells [6], and increased HSC number and function in aged mice [7, 8]. DR also increased HSC quiescence and enhanced repopulating capacity in 12 months old mice relative to *ad libitum* fed controls [9]. Similarly, very small embryonic-like stem cell number was increased in long-lived growth hormone deficient mice, compared to age-matched controls [10]. However, progenitor cells and HSCs have not been fully characterised in other genetic mouse models of longevity [3]; if stem cell dysfunction is an important driver of organismal aging [2], then one might predict that their function during aging would be preserved in long-lived mutants relative to control mice.

It is well established that hyperactivation of the mTOR signalling pathway can induce premature aging within HSCs [3, 11–13] and that activity of various components of the mTOR signalling pathway is increased within HSCs of aged mice [13]. In addition, rapamycin, which acts to inhibit the mTOR pathway, both extends lifespan in model organisms [14–16] and restores self-renewal capacity, hematopoiesis, *ex vivo* expansion and long-term reconstitution in aged murine HSCs [11, 13]. Similarly, rapamycin treatment restored autophagy activity in aged skeletal muscle satellite cells, attenuated senescence and increase expansion of aged satellite cells following engraftment [17]. We have previously reported that mice which are globally null for ribosomal protein S6 kinase 1 (*S6K1^−/−^*), a key downstream effector of mTOR [18], are long-lived and show extended healthspan relative to wild-type (WT) controls mice, including the preservation of naïve T-cell relative to memory T-cell subsets during aging [19]. Consequently, we predicted that HSC function would be enhanced in long-lived *S6K1^−/−^* mice relative to WT controls during aging. In order to test this prediction, we characterised gene expression profiles in isolated bone marrow (BM) c-Kit+ cells (HSCs and progenitor cells) of a number of candidate genes that have previously been linked to HSC function and maintenance [20–27], or whose expression has been shown to be altered within HSCs through modulation of the mTOR signalling pathway [11, 28]. Secondly, we investigated HSC function following transplantation challenge in young (12 weeks of age) and aged (80 weeks of age) WT control and *S6K1^−/−^* mice.

## RESULTS

**Figure 1:**
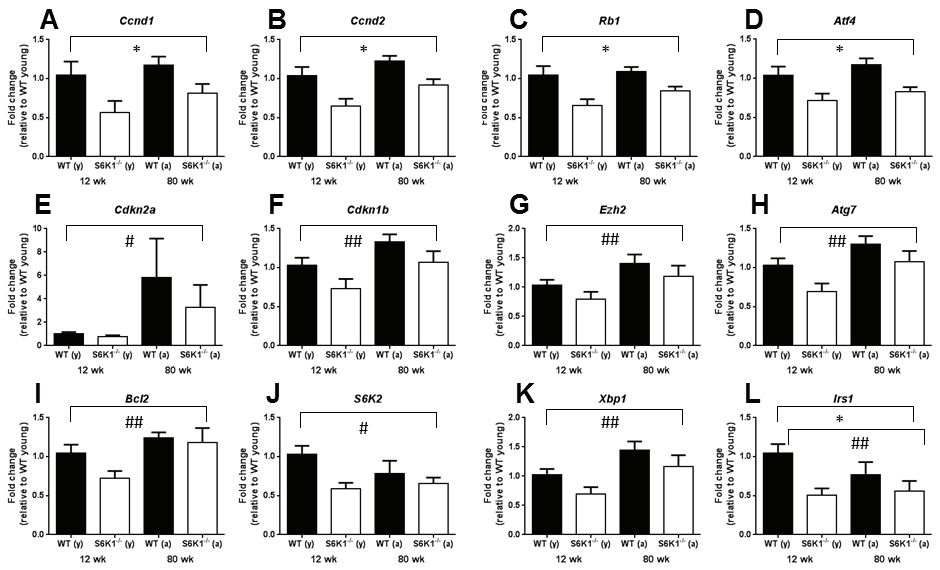
Gene expression levels of candidate genes within bone marrow derived c-Kit^+^ cells linked to HSC aging and function in young and aged WT and *S6K1^−/−^* mice Graphs show a significant genotype **A.**-**D.**, age **E.**-**K.** or both a significant genotype and an age effect **L.**. A significant interaction between genotype and age was observed for both *S6K2* **J.** and *Irs1* **L.**. Graphs display mean (±SEM) fold-change, all relative to the young WT group (female mice, *n* = 3-5; genotype effects * *P* < 0.05, age effects # *P* < 0.05, ## *P* < 0.01), with closed bars indicating WT mice and open bars indicating *S6K1^−/−^* mice. See also Tables S1 and S2.

To gain some potential insight in to how the absence of S6K1 may impact on stem cell function during aging, we isolated BM c-Kit^+^ cells (HSCs and progenitor cells) from young and aged WT and *S6K1^−/−^* mice and undertook gene expression profiling of candidate genes linked to HSC function and maintenance (Table S1 & S2). Using a multivariate approach, we identified several genes significantly affected by genotype, by age or that showed an interaction between both (Table S1). The mRNA expression levels of *Ccnd1, Ccnd2, Rb1, Atf4* and *Irs1* were all significantly reduced in *S6K1^−/−^* mice (Figure 1A-1D, 1L, Table S1), with age affecting the expression of *Cdkn2a, Cdkn1b*, *Ezh2*, *Atg7, Bcl2, S6K2, Xbp1 and Irs1* (Figure 1E-1K, 1L, Table S1). Only *S6K2* and *Irs1* showed a significant interaction between genotype and age, with the age-associated decrease in expression of both more pronounced in WT mice than in *S6K1^−/−^* mice.

**Figure 2:**
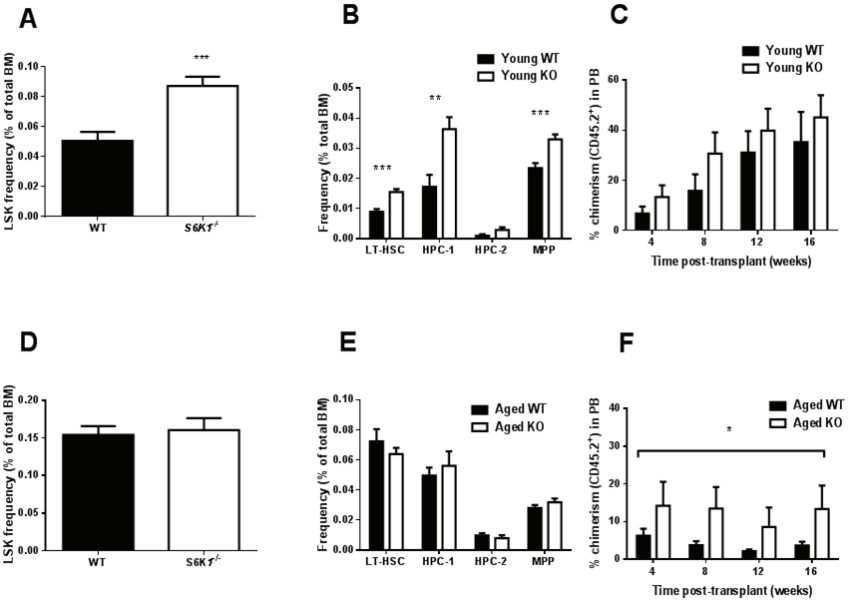
HSC frequency and function in young and aged WT and *S6K1^−/−^* mice **A.** LSK frequency (percentage of total BM), **B.** frequency (percentage of total BM) of LT-HSC, HPC-1, HPC-2 and MPP in young WT and *S6K1^−/−^* mice (*n* = 9-11, mixed gender). **C.** Percentage of chimerism (CD45.2^+^ cells) in peripheral blood (PB) from irradiated recipients transplanted with young WT or *S6K1^−/−^* LT-HSCs (donor cells female, recipients mixed gender, *n* = 6-9). The effect of genotype on chimerism was non-significant (*F* = 3.037, *P* = 0.088), although the effect of time was (*F* = 5.696, *P* = 0.002). **D.** LSK frequency and **E.** frequency of LT-HSC, HPC-1, HPC-2 and MPP in BM of aged WT and *S6K1^−/−^* mice (*n* = 7-15, mixed gender). **F.** Percentage of chimerism (CD45.2^+^ cells) in PB from irradiated recipients transplanted with aged WT or *S6K1^−/−^* LT-HSCs (donor cells female, recipients mixed gender, *n* = 6). A highly significant genotype effect was detected (*F* = 8.452, *P* = 0.006), but the effect of time was non-significant (*F* = 0.494, *P* = 0.688). (* *P* < 0.05, ** *P* < 0.01, *** *P* < 0.001). Values are mean±SEM, with closed bars indicating WT mice and open bars indicating *S6K1^−/−^* mice.

No genotype effects were detected in the cellularity of bone marrow (BM), spleen or thymus or in mature cell frequencies in BM of young mice (Figure S1A-S1D). Young *S6K1^−/−^* mice showed a greater frequency of stem/progenitors (LSK, lineage^−^, c-Kit^+^, Sca-1^+^) within BM compared to WT mice (Figure 2A). Young *S6K1^−/−^* mice also showed higher frequencies of long-term repopulating HSC (LT-HSC) (LSK, CD150^+^, CD48^−^), hematopoietic progenitor cells (HPC-1) (LSK, CD150^−^, CD48^+^) and multipotent progenitors (MPP) (LSK, CD150^−^, CD48^−^) in comparison to WT controls (Figure 2B). No difference in chimerism between genotypes (Figure 2C) was observed following transplantation of young female WT or *S6K1^−/−^* LT-HSC (CD45.2^+^) into irradiated recipients (CD45.1^+^), although a non-significant trend for an increase in percentage chimerism following transplantation with *S6K1^−/−^* LT-HSC cells was observed (*p* = 0.088). However, donor derived cell contribution indicated that *S6K1^−/−^* LT-HSC skewed mature cell frequency, with more T lymphoid cells at 16 weeks post transplantation (Figure S2A-S2E). In aged mice, genotype did not affect BM, spleen or thymus cellularity, or the frequency of BM mature cell types (Figure S3A-S3D). Similarly, the frequency of LSK cells, or HSC sub-populations within these LSK cells were unaffected by genotype (Figure 2D and 2E). However, following transplantation a greater percentage chimerism in mice transplanted with *S6K1^−/−^* LT-HSC cells (Figure 2F), strongly suggestive that HSC function was preserved during aging in *S6K1^−/−^* mice relative to WT controls. No genotypic difference in donor-derived mature cells within the BM of recipient mice was observed following transplantation (Figure S4A-S4D).

## DISCUSSION

Our preliminary findings in c-Kit^+^ cells indicate a significant reduction in the expression of *Ccnd1and Ccnd2* in *S6K1^−/−^* mice; genes that encode the G-1 phase regulators Cyclin D1 and 2. These findings suggest that reduced TOR signalling may be associated with greater quiescence within this cell population, as reported elsewhere [29]. Thus, genetic deletion of S6K1 may suppress geroconversion, that is a switch from reversible cell cycle arrest to senescence [30, 31], within HSCs during aging. Interestingly HSC quiescence has also been observed in mice following DR [9], suggesting that HSC quiescence may be a shared characteristic of different mouse models of slowed aging. However, it should be noted that the BM cells used in our study contained both HSCs and progenitor cells, and so an important next step will be to examine transcriptional changes solely within HSCs. Interestingly, the expression levels of insulin receptor substrate 1 (*Irs1*) was significantly lower in *S6K1^−/−^* cells relative to WT cells (Figure 2L). IRS1 is a key adaptor protein within the insulin/IGF-1 signalling pathway, and we have previously reported that *Irs1^−/−^* mice have extended lifespan and healthspan relative to WT mice [32]. Whether preserved HSC function is a conserved phenotype that is found across different long-lived genetic mutant mice has yet to be established. We observed no age-associated increase in either erythropoiesis or myelopoiesis in BM of young or aged *S6K1^−/−^* mice, in contrast to what has been reported in DR mice [9]. In line with our original prediction, HSCs derived from aged female *S6K1^−/−^* mice showed a greater percentage engraftment following transplantation, in line with studies examining HSC function in aged mice following rapamycin treatment [11, 13]. Importantly, no difference in engraftment, relative to WT mice, was observed when HSCs from young *S6K1^−/−^* mice were transplanted in to irradiated hosts, suggesting that the loss of S6K1 specifically slowed an age-associated deterioration in HSC function. The findings of this study provide compelling evidence that the functional decline of HSCs during aging is ameliorated in long-lived mTOR mutant mice. Consequently, these observations further underscore the potential importance of mTOR signalling to HSC aging, and may provide new insights into the potential linkage between HSC ageing and organismal lifespan and healthspan [13, 28, 29, 33]. The clear challenge now following on from these initial findings is to now fully characterise HSCs from these mice in order to help identify the mechanisms underpinning the observed preservation of HSC function during aging. For example, a critical next experiment will be to investigate engraftment following transplantation of male-derived *S6K1^−/−^* LT-HSCs; male *S6K1^−/−^* mice show evidence of extended healthspan but are not long-lived relative to male WT mice [19].

## MATERIALS AND METHODS

### Ethics statement

Investigation has been conducted in accordance with the ethical standards and according to the Declaration of Helsinki and according to national and international guidelines and has been approved by the authors’ institutional review board. All procedures followed the 1986 UK Home Office Animals Procedures Act (PPL: 60/4492 and 70/7438).

*S6K1* wild-type (WT) and *S6K1* global null (*S6K1^−/−^*) mice were bred and maintained following previously described protocols [19, 34]. B6SJL mice expressing CD45.1 were bred and maintained according to standard procedures (Beatson Institute for Cancer Research, Glasgow, UK). WT and *S6K1^−/−^* animals were fasted overnight and culled by cervical dislocation. Blood was harvested in EDTA treated tubes (Sarstedt AG & Co, Germany) and leg bones (femur and tibia), spleen and thymus were harvested in PBS supplemented with 2% FBS (PBS/2%FBS) on ice. Mice were culled at either 12 weeks (12.86±0.07 wks; referred to as young in text) or 80 weeks of age (82.83±0.71 wks; referred to as aged in text). B6SJL mice were used for the transplantation assays at 10 weeks of age (10±0.50 wks).

Bones were subsequently crushed using a pestle and mortar in PBS/2%FBS and spleen/thymus were mashed using a 5mL syringe in a petri dish and all cell suspensions were filtered through a 70μm mesh in PBS/2%FBS. Cellularity was assessed in bone marrow (BM), spleen and thymus cells using an automated cell counter. Briefly, the number of cells was quantified using the Guava ViaCount reagent using a 1:10 dilution and the Guava easyCyte flow cytometer (Millipore UK Ltd, Hertfordshire, UK). Cellularity per mL was multiplied by the total volume of cell suspension to get the absolute number of white blood cells (WBC) per harvest.

BM, spleen and thymus cell suspensions were spun in a flow cytometry (FACS) tube at 400x*g* for 5 min with 1×10^5^ or 1×10^6^ cells and incubated with Fc block for 5 min on ice (excluding cells stained with an antibody against CD16/CD32). Cells were subsequently stained with cell surface markers in PBS/2%FBS and incubated on ice for 20 min before washed in PBS/2%FBS. Cells stained with antibodies against biotin were subsequently stained with streptavidin, incubated for 20 min on ice and washed in PBS/2%FBS before FACS analysis. Blood samples were incubated with the antibody mix for 20 min at room temperature (RT) with subsequent addition of red blood cell (RBC) lysis buffer (Dako UK Ltd, Cambridgeshire, UK) as per the manufacturer's protocol. After 10 min at RT in the lysis buffer, FACS analysis was carried out immediately. Cell surface antibodies were purchased from BD Biosciences (Oxford, UK), eBioscience Ltd (Hatfield, UK) or BioLegend (London, UK) unless otherwise stated. Antibodies used were against Gr-1, CD11-b, CD19, Ter-119, CD4, CD8a, CD44, CD45RB, CD5, B220, c-Kit, Sca-1, CD150, CD48, CD16/32, CD127 and CD34. FACS analysis was carried out using a LSRII, FACS Canto II and cells were sorted using a FACS Aria (BD Biosciences). FACS analysis was carried out using FlowJo software (Ashland, OR, USA) using unstained and single colour controls. All gating strategies used are displayed (Figure S5).

B6SJL mice were irradiated with 7Gy 24 hours prior to transplant. Mice were treated with Baytril for 2 weeks post-transplant. 100 LT-HSC (Lineage^−^, c-Kit^+^, Sca-1^+^, CD150^+^, CD48^−^) from donor mice (CD45.2^+^) were sorted into a cell suspension of CD45.1^+^ support BM (200,000 cells) for intravenous injection per irradiated recipient mouse. Animals were bled every 4 weeks post-transplant to assess chimerism using FACS. After 16 weeks post-transplant, animals were culled and BM harvested to examine chimerism. Chimerism was assessed using the percentage of donor derived cells (CD45.2^+^) in the PB and BM of recipient mice. If the percentage chimerism was < 1% in the peripheral blood then we assumed failed engraftment and this data was excluded from all subsequent analysis (See Figure S6 for a schematic representation of the experiment).

c-Kit^+^ cells from BM were isolated using positive microbead separation (Miltenyi Biotec Ltd, Surrey, UK). After isolation, RNA was extracted (Qiagen Ltd, Manchester, UK) and RNA concentration and quality assessed using a NanoDrop and Agilent bioanalyser (Agilent technologies LDA Ltd, Cheshire, UK). Total RNA (290ng) from each sample was synthesised into cDNA (Life Technologies Ltd, Paisley, UK) and subsequently pre-amplified using Taqman™ pre-amplification master mix with Taqman™ probes of interest and protocol according to standard procedures. Pre-amplified cDNA was run on a 48.48 Fluidigm chip as per standard procedures (Fluidigm UK Ltd, Cambridge, UK) and data analysed using biomark software (Fluidigm UK Ltd). The ΔΔCT method was used to assess differences between groups using the WT young group as the calibrator. Taqman probes with assay ID mentioned in this study can be viewed in Table S1.

Data was analysed using GraphPad Prism version 6.00 (GraphPad Software, La Jolla Ca, USA, www.graphpad.com) and SPSS version 22 (SPSS Inc., Chicago, Il, USA). Student's unpaired *t*-test and general linear models (GLM) were used to assess statistically significant differences between groups.

## SUPPLEMENTARY MATERIAL FIGURES AND TABLES


