# Supplementary material for: Evidence that hematopoietic stem cell function is preserved during aging in long-lived S6K1 mutant mice
Source: Oncotarget. 2016 Apr 13;7(21):29937–43. doi: 10.18632/oncotarget.8729 (PMC5058654; doi:10.18632/oncotarget.8729)
Supplement: Supplementary file 1 [file oncotarget-07-29937-s001.pdf]

Evidence that hematopoietic stem cell function is preserved during aging in long-lived S6K1 mutant mice

Supplementary Material

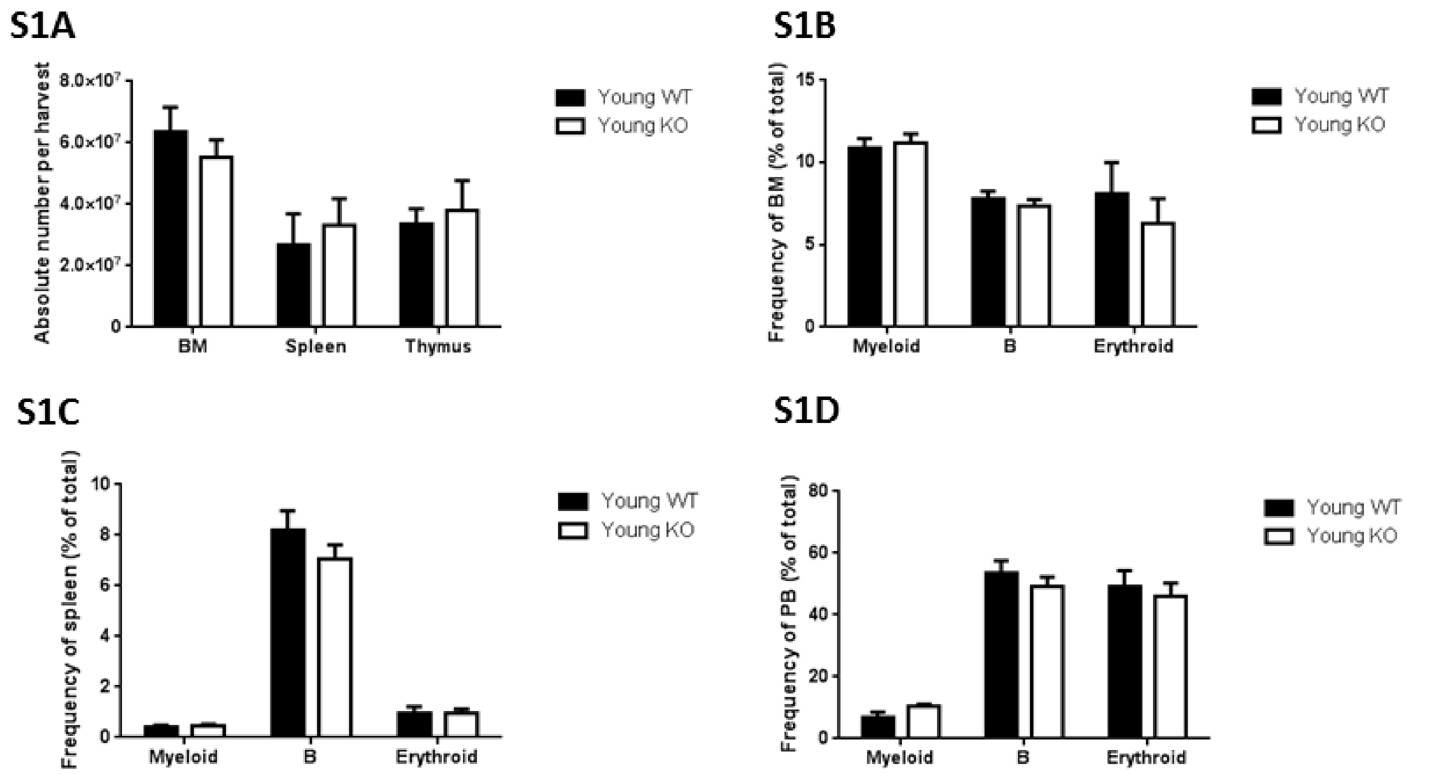

Figure S1A-D: Young mice. S1A. Cellularity per harvest in the bone marrow (BM), spleen and thymii of WT and *S6K1*$^{-/-}$ mice (n = 9-11, mixed gender). S1B. Frequency of myeloid (Gr-1+, CD11-b+), B (CD19+) and Erythroid (Ter-119+) cells in BM of (n = 9-11, mixed gender). S1C. Frequency of myeloid (Gr-1+, CD11-b+), B (CD19+) and Erythroid (Ter-119+) cells in spleen of (n = 9-11, mixed gender). S1D. Frequency of myeloid (Gr-1+, CD11-b+), B (CD19+) and Erythroid (Ter-119+) cells in PB of (n = 9-11, mixed gender).

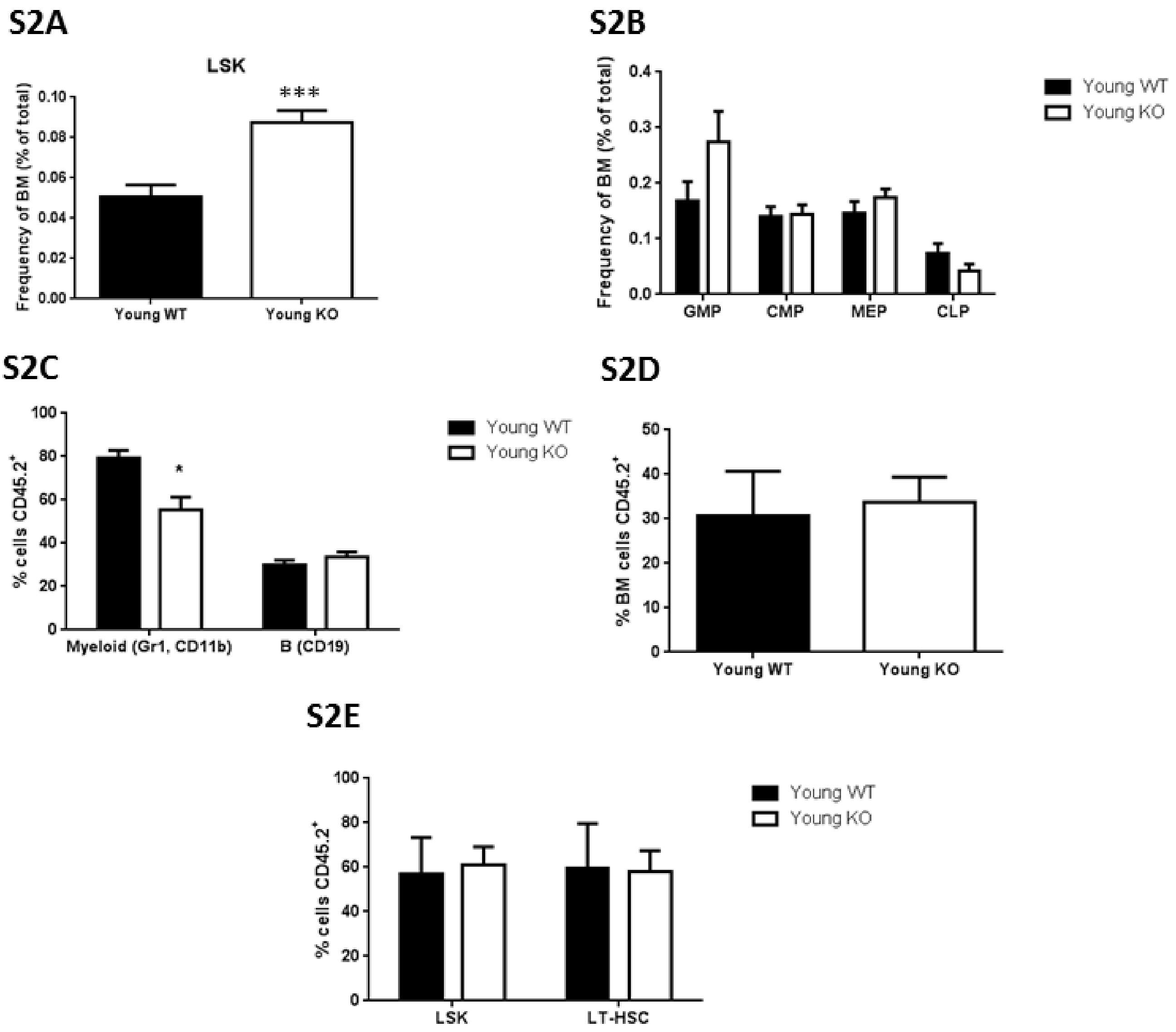

Figure S2A-E: Young mice. S2A. Frequency of cells positive for lineage-, c-Kit+, Sca-1+ cells (LSK) in the BM of WT and *S6K1*$^{-/-}$ mice (n = 9-11, mixed gender) *** *P*<0.001. S2B. Frequency of cells positive for progenitor cells (GMP (CD34^{+}, CD16/32^{+}), CMP (CD34^{+}, CD16/32^{-}), MEP (CD34^{-}, CD16/32^{-}) and CLP (CD 127^{+}) in the BM of WT and *S6K1*$^{-/-}$ mice (n = 9-11, mixed gender). S2C. Percentage of myeloid or B lymphoid cells donor derived (CD45.2^{+}) in the BM of transplanted mice (n=5-6 per group). S2D. Percentage of CD45.2^{+} in the BM of recipient mice transplanted with young WT or *S6K-1*$^{-/-}$ cells (donor cells female, recipients mixed gender, n=5-6 per group). S2E. Percentage of LSK and LT-HSC donor derived (CD45.2+) in BM of 16 week post-transplant recipient mice (n=5-6 per group).

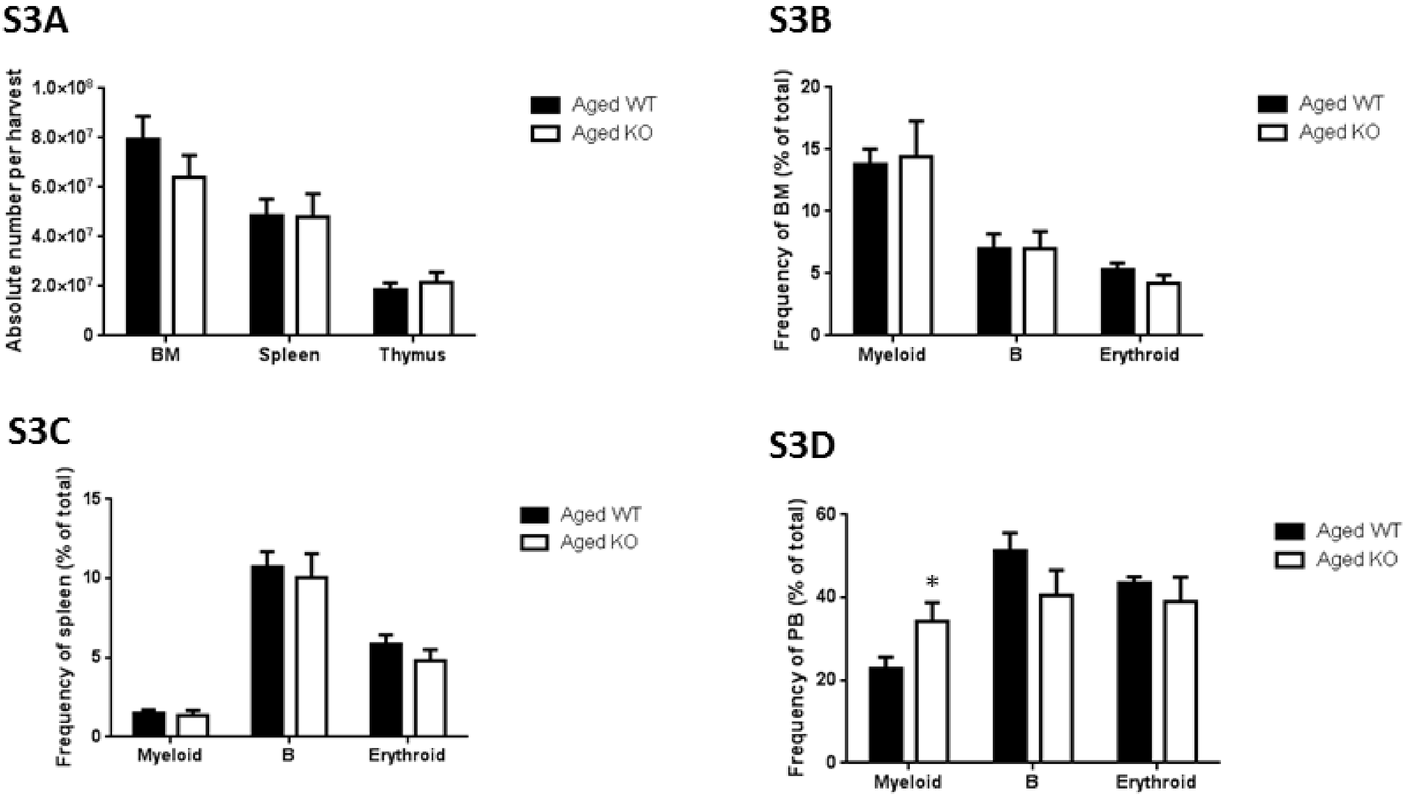

Figure S3A-D: Aged mice. S3A. Cellularity per harvest in the BM, spleen and thymii of aged WT and *S6K1*$^{-/-}$ mice (n = 11-18, mixed gender). S3B. Frequency of myeloid (Gr-1+, CD11-b+), B (CD19+) and Erythroid (Ter-119+) cells in BM of aged WT and *S6K-1*$^{-/-}$ mice (n = 11-15, mixed gender). S3C. Frequency of myeloid (Gr-1+, CD11-b+), B (CD19+) and Erythroid (Ter-119+) cells in spleen of aged WT and *S6K-1*$^{-/-}$ mice (n = 11-15, mixed gender). S3D. Frequency of myeloid (Gr-1+, CD11-b+), B (CD19+) and Erythroid (Ter-119+) cells in the PB of aged WT and *S6K-1*$^{-/-}$ mice (n = 5-11, mixed gender * P<0.05).

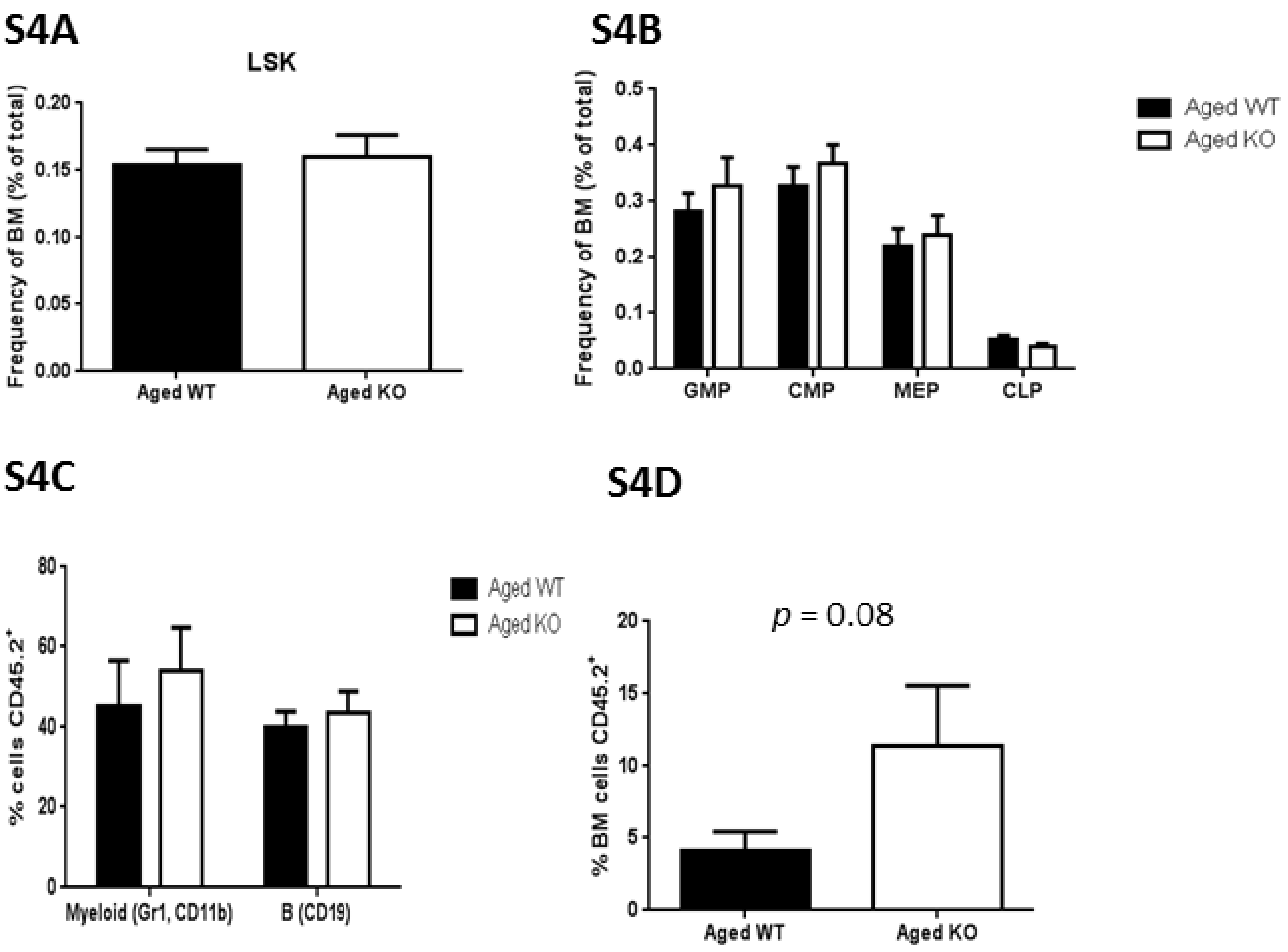

Figure S4A-D: Aged mice. S4A. Frequency of cells positive for lineage-, c-Kit+, Sca-1+ cells (LSK) in the BM of aged WT and *S6K1*$^{-/-}$ mice (n = 7-15, mixed gender). S4B. Frequency of cells positive for progenitor cells (GMP, CMP, MEP and CLP) in the BM of WT and *S6K1*$^{-/-}$ aged mice (n =7-14, mixed gender). S4C. Percentage of myeloid or B lymphoid cells donor derived (CD45.2^{+}) in the BM of transplanted mice (n=6 per group).S4D. Percentage of CD45.2^{+} in the BM of recipient mice transplanted with aged WT or *S6K-1*$^{-/-}$ cells (donor cells female, recipients mixed gender, n=6 per group).

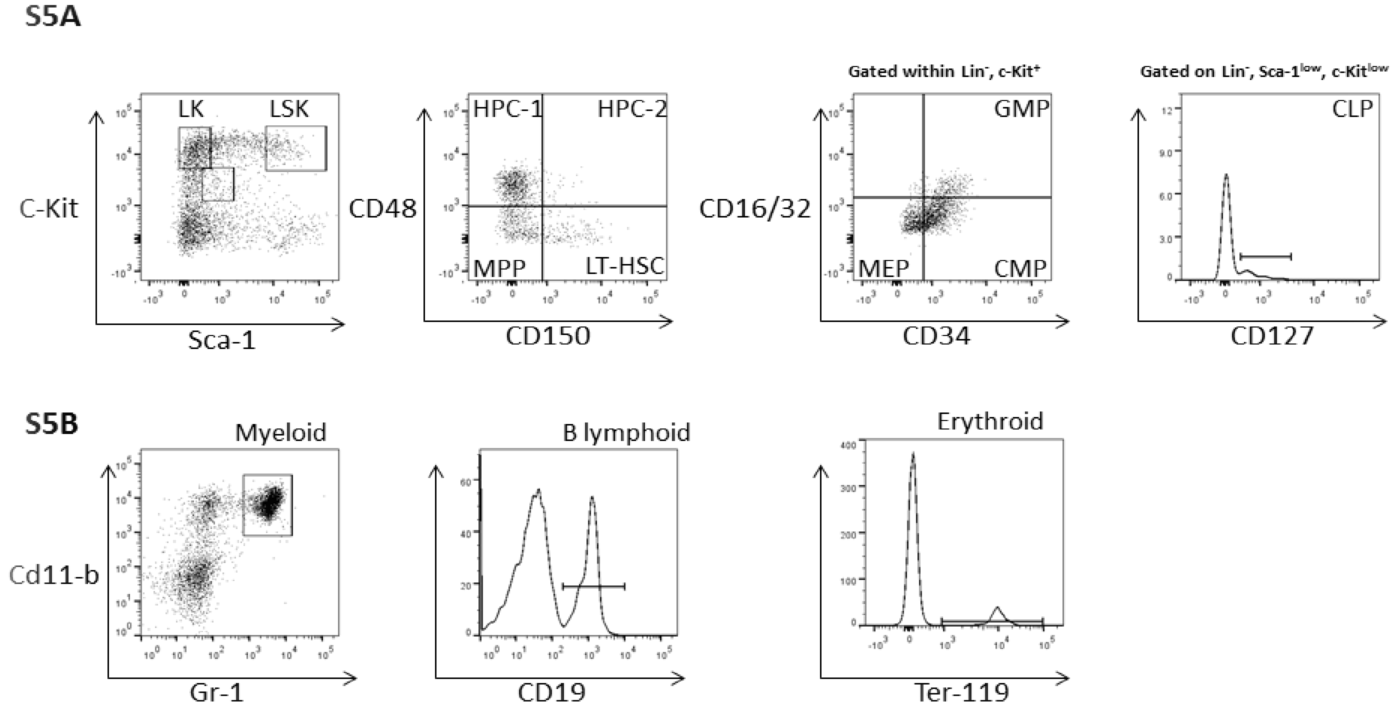

Figure S5A and B. S5A shows a representative BM plot displaying cell distribution for Sca-1 and c-Kit within lineage negative cells. Within the LSK population the middle dot plot shows representative staining for CD150 and CD48 to identify HSC sub populations. The right dot plot displays CD34 and CD16/32 staining within the LK population to identify GMP, MEP and CMP populations. Within lineage negative, c-Kit^{low} and Sca-1^{low} cells, cells were identified as CLP based on CD127^{+} expression. S5B shows a representative BM plot displaying cell distribution for Gr-1 and Cd11-b to identify myeloid cells. The middle and right histograms respectively display representative plots displaying CD19^{+} cells and Ter-119^{+} cells displaying gating strategy for B lymphoid and erythroid cells respectively.

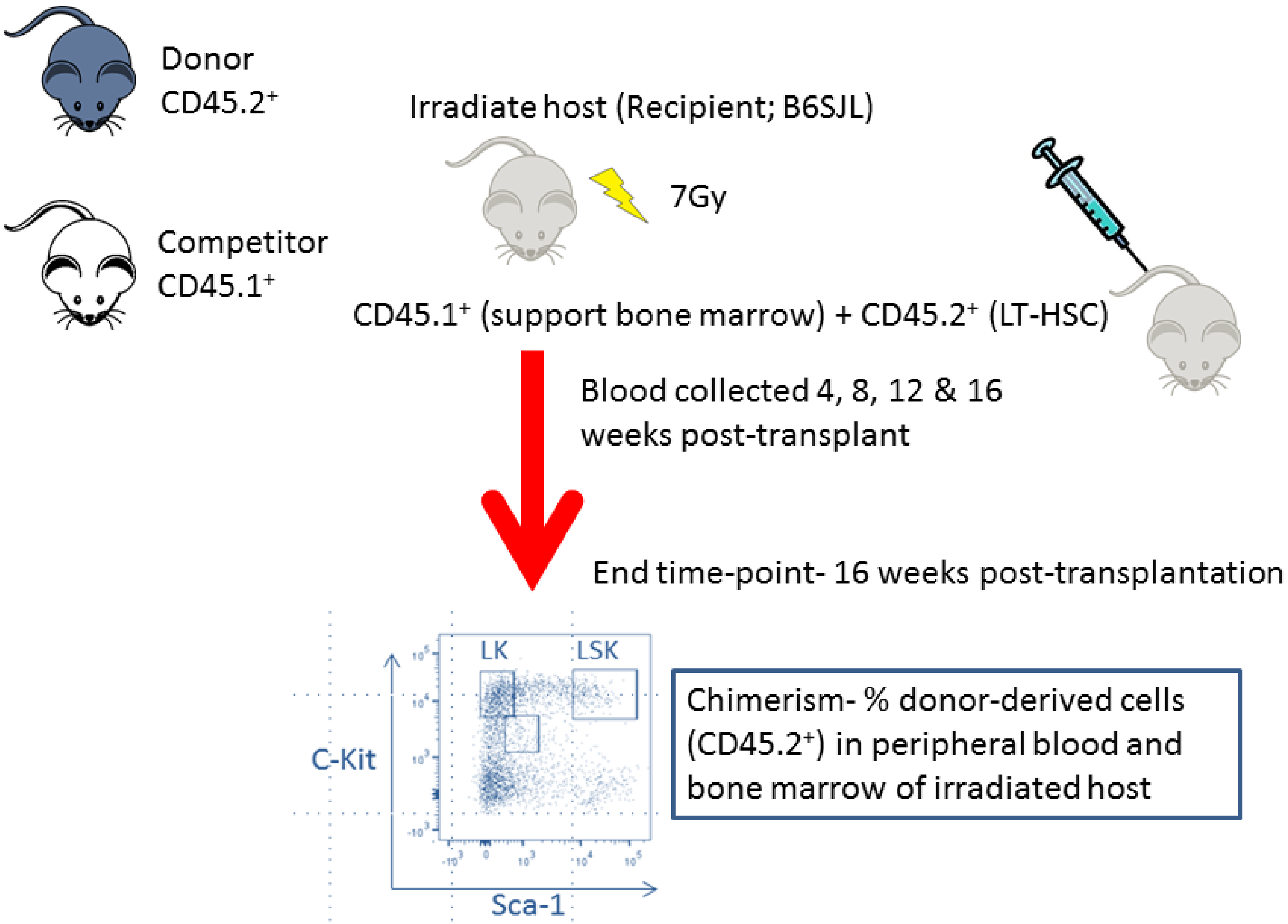

Figure S6. Schematic representation of the transplantation experimental protocol employed for both young and aged mice.

Table S1. mRNA levels of candidate genes linked to HSC function in c-Kit^{+} cells. All data analysed using a general linear model, where all non-significant (NS) interaction effects (p>0.05) were subsequently removed from each analysis in order to obtain the best-fitted model in each case. Genotype effects (WT vs *S6K1*$^{-/-}$) and age effects (young 12 wk vs. old 80 weeks). All female mice, where n=3-5.

Gene symbol	Genotype effect	Age effect	Interaction effect
**Cdkn2a**	0.502	**0.046**	NS
**Bmi1**	0.192	0.335	NS
**Cdkn1a**	0.673	0.091	NS
**Ccdnd1**	**0.011**	0.216	NS
**Ccnd2**	**0.033**	0.050	NS
**Cdkn1b**	0.355	**0.009**	NS
**Cknnb1**	0.761	0.153	NS
**Rb1**	**0.045**	0.698	NS
**Eprs1**	0.272	0.405	NS
**Dnajb9**	0.610	0.303	NS
**Ddit4**	0.725	0.725	NS
**Ezh2**	0.981	**0.003**	NS
**Ddit3**	0.605	0.137	NS
**Cxcl1**	0.244	0.098	NS
**Fox01**	0.203	0.660	NS
**Atf4**	**0.045**	0.572	NS
**Atg7**	0.298	**0.004**	NS
**Bcl2**	0.930	**0.004**	NS
**Ccdn3**	0.070	0.206	NS
**Cxcl5**	0.159	0.471	NS
**Dppa5a**	0.248	0.943	NS
**Fox03a**	0.625	0.447	NS
**Gata2**	0.174	0.547	NS
**Gfi1**	0.653	0.408	NS
**Gsk3b**	0.183	0.433	NS
**Irs1**	**0.020**	**0.005**	**0.040**
**Irs2**	0.254	0.464	NS
**Mc11**	0.750	0.328	NS
**Mi67**	0.718	0.691	NS
**Nfe212**	0.234	0.915	NS
**Nrf1**	0.367	0.873	NS
**Pck3r1**	0.238	0.880	NS
**Pik3ca**	0.407	0.094	NS
**Psmd11**	0.420	0.110	NS
**Pten**	0.157	0.403	NS
**Rps6kb2**	0.187	**0.013**	**0.010**
**Sod1**	0.754	0.750	NS
**Sod2**	0.576	0.922	NS

**Tgfb1**	0.224	0.300	NS
**Trp53**	0.184	0.276	NS
**Ulk1**	0.586	0.118	NS
**Xbp1**	0.278	**0.001**	NS
**Sk2b3**	0.655	0.360	NS
**18S (control)**	0.462	0.133	NS

Table S2. List of probes used for qPCR analysis. Table displays gene name and assay ID of each Taqman™ probe used.

**Gene name**	**Assay ID**
18S	Mm03928990_g1
Ccnd1	Mm00432359_m1
Ccnd2	Mm00438070_m1
Ccnd3	Mm01612362_m1
Cdkn2a	Mm00494449_m1
Cdkn1a	Mm00432448_m1
Cdkn1b	Mm00438168_m1
Mki67	Mm01278617_m1
Rb1	Mm00485586_m1
Ezh2	Mm00468464_m1
Bmi1	Mm03053308_g1
Gata-2	Mm00492301_m1
Gfi1	Mm00515853_m1
Fox01	Mm00490671_m1
Fox03	Mm01185722_m1
Atf4	Mm00515324_m1
Xbp1	Mm00457357_m1
Ddit3	Mm00492097_m1
Bcl2	Mm00477631_m1
Mcl1	Mm01257351_g1
Irs1	Mm01278327_m1
Irs2	Mm03038438_m1
Pik3ca	Mm00435673_m1
Pik3r1	Mm01282781_m1
Sod1	Mm01344233_g1
Sod2	Mm01313000_m1
Tgfb	Mm01178820_m1
Pten	Mm00477208_m1

S6K1	Mm01310033_m1
S6K2	Mm00445440_m1
Eprs	Mm01315474_m1
Trp53	Mm01731290_g1
Sh2b3	Mm00833471_m1
Cxcl1	Mm04207460_m1
Cxcl5	Mm00436451_g1
Ulk1	Mm00437238_m1
Nrf1	Mm01135606_m1
Nfe2l2	Mm00477784_m1
Ddit4	Mm00512504_g1
Dnajb9	Mm01622956_s1
Dppa5	Mm01171664_g1
Psmd11	Mm00780758_sH
Ctnnb1	Mm00483039_m1
Gsk3b	Mm00444911_m1
Atg7	Mm00512209_m1
Gene name	Assay ID
18S	Mm03928990_g1
Ccnd1	Mm00432359_m1
Ccnd2	Mm00438070_m1
Ccnd3	Mm01612362_m1
Cdkn2a	Mm00494449_m1
Cdkn1a	Mm00432448_m1
Cdkn1b	Mm00438168_m1
Mki67	Mm01278617_m1
Rb1	Mm00485586_m1
Ezh2	Mm00468464_m1
Bmi1	Mm03053308_g1
Gata-2	Mm00492301_m1
Gfi1	Mm00515853_m1
Fox01	Mm00490671_m1
Fox03	Mm01185722_m1
Atf4	Mm00515324_m1
Xbp1	Mm00457357_m1
Ddit3	Mm00492097_m1
Bcl2	Mm00477631_m1
Mcl1	Mm01257351_g1
Irs1	Mm01278327_m1
Irs2	Mm03038438_m1
Pik3ca	Mm00435673_m1
Pik3r1	Mm01282781_m1
Sod1	Mm01344233_g1
Sod2	Mm01313000_m1

Tgfb	Mm01178820_m1
Pten	Mm00477208_m1
S6K1	Mm01310033_m1
S6K2	Mm00445440_m1
Eprs	Mm01315474_m1
Trp53	Mm01731290_g1
Sh2b3	Mm00833471_m1
Cxcl1	Mm04207460_m1
Cxcl5	Mm00436451_g1
Ulk1	Mm00437238_m1
Nrf1	Mm01135606_m1
Nfe2l2	Mm00477784_m1
Ddit4	Mm00512504_g1
Dnajb9	Mm01622956_s1
Dppa5	Mm01171664_g1
Psmd11	Mm00780758_sH
Ctnnb1	Mm00483039_m1
Gsk3b	Mm00444911_m1
Atg7	Mm00512209_m1
